# Insights into the role of tumor abnormal protein in early diagnosis of cancer: A prospective cohort study

**DOI:** 10.1097/MD.0000000000019382

**Published:** 2020-03-13

**Authors:** Lu-Xi Li, Bin Zhang, Rui-Zhi Gong

**Affiliations:** a Department of Ophthalmology, Xi’an No 3 Hospital, The Affiliated Hospital of Northwest University; b Department of Hepatobiliary Surgery, Xijing Hospital, The Air Force Medical University; c Department of Oncology, Xi’an International Medical Center, Xi’an, Shaanxi, China.

**Keywords:** tumor abnormal protein, diagnosis of cancer, cohort study, oncogenic transformation, alpha fetoprotein, carbohydrate antigen 19-9

## Abstract

The aim of this study was to evaluate the clinical use of tumor abnormal protein (TAP) in the diagnosis of different cancers.

Totally 394 patients were divided into 4 groups, namely 100 healthy volunteers, 167 patients with cancer, 20 subjects with precancerous lesions, and 107 subjects with benign lesions. TAP was detected in 4 groups of research subjects using a TAP testing kit and examination system. We correlated TAP levels with a wide variety of clinical indicators as well as established cancer markers, including alpha fetoprotein (AFP) and carbohydrate antigen 19-9 (CA19-9). Besides, the changes of TAP level in 51 patients with liver cancer before and after surgery, and overall survival of patients with high or low TAP expression in pancreatic, gallbladder, bile duct, and liver cancers were analyzed.

Statistically significant difference was observed in the TAP-positive ratio among subjects with cancer (79.6%) and precancerous lesions (45.0%) compared to the healthy volunteers (4.0%). TAP expression in different cancers was characterized by high sensitivity (79.64%), specificity (89.87%), positive and negative predictive value (85.25% and 85.71%), overall compliance rate (85.53%) but low omission and mistake diagnostic rate (20.36% and 10.13%), Youden index (0.6951). In addition, there was no significant difference among patients with different types of cancer (*χ*^2^ = 2.886, *P* = .410), and TAP expression was shown to be correlated with AFP in liver cancer (*P* = .034) but not with CA19-9 in pancreatic cancer (*P* = .241). Moreover, the overall survival of patients with low expression of TAP in pancreatic, gallbladder, bile duct, and liver cancers were significantly higher than of patients with high expression of TAP. Compared with the preoperative patients with cancer, TAP levels decreased dramatically among postoperative subjects (*P* < .001).

In summary, TAP might hold promise in serving as universal indicator for the diagnosis of different cancers.

## 1 Introduction

Cancer, as a multifactorial disease, remains the greatest challenge in the modern health care all over the world. It has been recognized that the majority of patients with cancer were diagnosed at advanced stage, which made radical surgery impossible and treatment efficacy poor.^[1]^ On these bases, it is essential to detect cancer in the early stage where the universal cancer biomarkers hold considerable promise. As diagnostic indicator, it is supposed to distinguish healthy candidates from diseased patients within a wide variety of different cancers.^[2]^ Recently, accumulating studies have explored the universal usefulness of some biomarkers in human tumors.^[3–5]^

Glycoproteins with abnormal sugar chains, which exist in the cell membrane, have been implicated in the carcinogenesis.^[6]^ It occurs in different cancers and has been identified to be involved in cancer progression, metastasis, and the survival rate of patients.^[7,8]^ Numerous tumor-associated glycans are found at low level in normal tissues and at high level in tumors.^[9]^

Tumor abnormal protein (TAP), as a collective term for glycoproteins, is regarded as the common feature of malignant tumors.^[10,11]^ TAP could be easily detected in the peripheral blood once their levels achieve a certain threshold, which makes little damage to the patients and renders great convenience in diagnosis. Lan et al^[1]^ have clarified that most patients with early stage gastric cancer were TAP-positive. Similarly, TAP expression has also been shown increased in bladder cancer^[12]^ and colorectal cancer.^[13]^ However, there is little report on the comprehensive study of TAP role among different cancers.

This study was designed to clarify the potential of TAP as cancer marker with comprehensive study of clinical patients. On this basis, we attempted to make further step by investigating the potential role of TAP in other cancers, for example, liver, pancreatic, gallbladder, and bile duct, which to our knowledge have not been clarified; the performance of TAP compared with other established cancer marker, such as alpha fetoprotein (AFP), and carbohydrate antigen 19-9 (CA19-9); the potential of combining TAP with other marker in cancer prognosis.

## 2 Materials and methods

### 2.1 Patients

A total of 394 patients (201 males and 193 females, 22–78 years old) who were treated in the Department of Hepatobiliary Surgery from May to September in 2016 were enrolled in the present study. Among them, there were 100 healthy volunteers, 167 patients with cancer, 20 precancerous lesions, and 107 benign lesions. TAP was detected in 4 groups of research subjects. For the cancer group, there were 34 cases with pancreatic cancer, 73 liver cancer, 30 gallbladder cancer, and 30 bile duct cancer. For the precancerous lesion group, there were 4 cases of gallbladder intraepithelial neoplasia, 13 gallbladder polyps, and 3 pancreatic intraductal papillary mucinous tumor. For the benign lesion group, there were 82 bile duct stone, 5 hepatic cyst, 10 hepatic hemangioma, and 10 splenomegaly. Exclusion criteria: patients with immunodeficiency, hepatitis, diabetes, tuberculosis, and other diseases. All patients were pathologically diagnosed after surgery. All patients signed an informed consent before treatment, and this study was approved by the ethics committee. The clinical data of the patients are shown in Table 1.

**Table 1:**
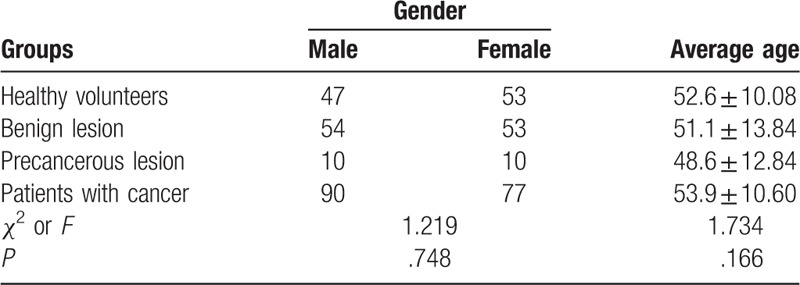
Baseline characteristics of subjects in 4 groups.

### 2.2 Detection of TAP

Peripheral blood was collected from healthy volunteers, all patients (1 day before surgery) and patients with cancer (1 week after surgery). TAP was detected using a TAP testing kit and examination system (Zhejiang Ruisheng Medical Technology, Ltd, Cixi, China) as previously reported.^[1]^ Samples were regarded as TAP positive only if the condensed particles meet the criteria: having a single condensate with an area of ≥225 μm^2^ or having 3 or more condensates with an area of 121 to 225 μm^2^, having 2 condensates with an area of 121 to 225 μm^2^ or having 3 or more condensates with an area of 81 to 121 μm^2^. Samples were confirmed as TAP-negative when there was no condensate, or condensates with an area of <81 μm^2^ or 2 or less condensates with an area of 81 to 121 μm^2^.

### 2.3 Detection of AFP and CA19-9

The serum AFP and CA19-9 level that analyzed in a gamma counter (Cobra II; Packard, Meriden, CT) were collected from medical record.

### 2.4 Statistical analysis

Statistical analysis was performed using the Statistical Package for the Social Sciences (SPSS) version 23.0 (SPSS Inc, Chicago, IL). The Pearson Chi-squared test and the Fisher exact test, if appropriate, were used to compare gender and the positive rate of TAP among groups. Analysis of variance was used to compare the age among groups. The correlation between TAP and established markers, for example, AFP and CA19-9 was analyzed. Preoperative and postoperative measurements were presented with mean ± standard deviation (*x* ± *s*) and compared with paired *t* test. For all comparisons, *P* < .05 was considered with statistical significance.

## 3 Results

### 3.1 High positive rate of TAP expression was observed in cancer lesions compared with normal samples

According to previous studies, aberrant glycosylation has been observed in nearly all types of cancers and is associated with tumor progression, metastasis, and the survival rate of patients.^[7,8]^ TAP is a collective term for glycoproteins which constitute most clinical tumor markers. Numerous studies have been reported to assess the prognostic value of TAP in different cancers, for example, gastric, bladder, and colorectal cancer. Therefore, to find out the function of TAP in other cancers such as liver, pancreatic, gallbladder, and bile duct cancer, a total of 394 clinical subjects were analyzed in this study.

**Figure 1:**
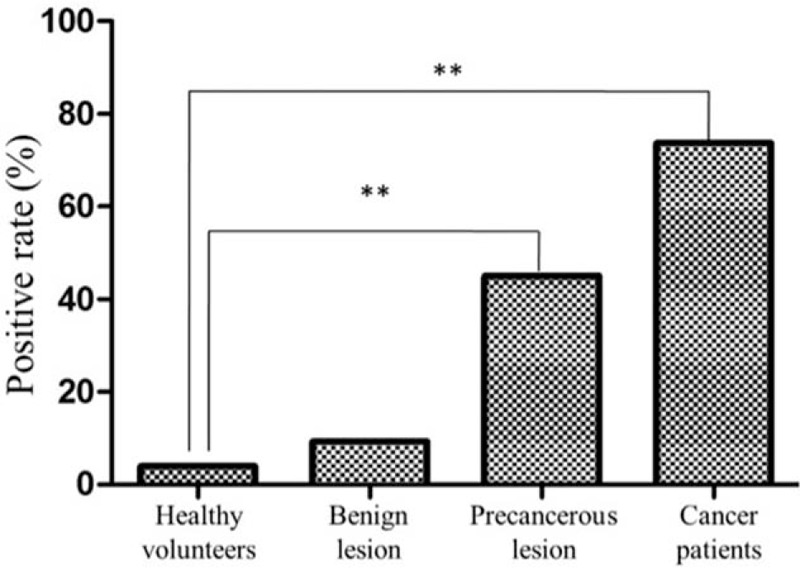
The positive rate of high tumor abnormal protein expression in different groups (healthy volunteers, subjects with benign lesion, precancerous lesion, and patients with cancer). ^∗∗^*P* < .01.

Totally 394 participants were divided into 4 different groups, including 100 healthy volunteers, 167 patients with cancer, 107 subjects with benign lesion, and 20 subjects with precancerous lesion. The baseline characteristics, for example, age and gender, are listed in Table 1, indicating no significant difference in the configuration among 4 groups (*P* = .748 for gender, *P* = .166 for age). Furthermore, the positive rate of TAP expression in 4 different groups were shown to be 4.0%, 9.3%, 45.0%, and 79.6% for healthy volunteers, benign lesions, precancerous lesions, and patients with cancer, respectively (Table 2). Compared with samples from health volunteers, the expression of TAP was found dramatically enhanced in cancer-related lesions, which could be evidenced by the much higher positive rate (*P* < .01: precancerous lesions, *P* < .01: patients with cancer) (Fig. 1). These results suggest that TAP has a higher positive expression rate in cancer lesions.

**Table 2:**
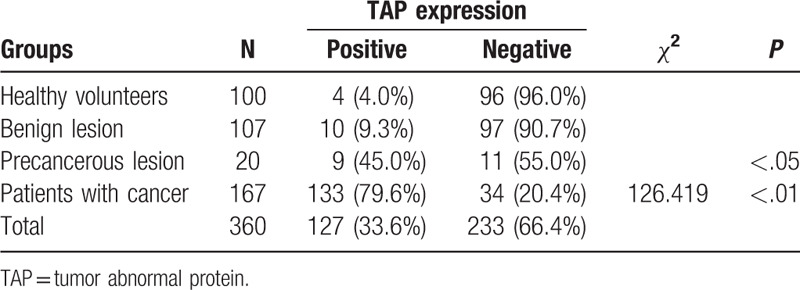
The expression of TAP in subjects of 4 different groups.

### 3.2 TAP expression holds promise in cancer diagnosis as an indicator

We evaluated the potential of TAP expression in diagnosis of cancer. A variety of indicators were determined on the basis of TAP expression, including sensitivity, specificity, omission diagnostic rate, mistake diagnostic rate, Youden index, positive predictive value, negative predictive value, and overall compliance rate, which represented the early diagnostic indicators of cancer. From our results, it can be found that TAP expression in different cancers was characterized by high sensitivity (79.64%), specificity (89.87%), positive and negative predictive value (85.25% and 85.71%), and overall compliance rate (85.53%) but low omission and mistake diagnostic rate (20.36% and 10.13%), and Youden index (0.6951) (Table 3). Collectively, these suggested that TAP represents a promising indicator for universal cancer detection and diagnosis.

**Table 3:**
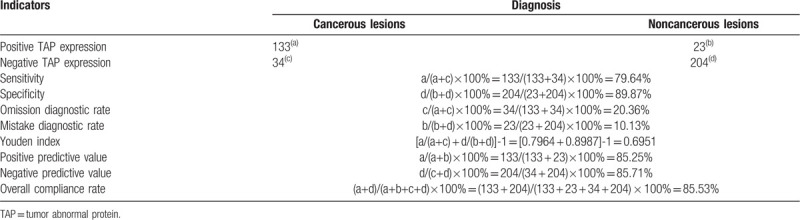
Evaluation indicators of TAP detection for early detection and diagnosis of cancer.

### 3.3 High positive rate of TAP expression was found in all different cancers

According to the above results, we were confident with the difference of TAP between cancer and noncancer samples. Furthermore, we were interested to elaborate the variance of TAP among different cancers. To this end, 4 types of cancer were investigated in the present study (Table 4), including liver cancer (73 cases), pancreatic cancer (34 cases), gallbladder cancer (30 cases), and bile duct cancer (30 cases). To our knowledge, no study was reported on the expression of TAP in these cancers. As a result, the positive rate of TAP expression was determined to be 70.6%, 79.5%, 83.3%, and 86.7% for pancreatic, liver, gallbladder, and bile duct cancer, respectively (Table 4). Moreover, there was no statistically significant difference among 4 types of cancer (*χ*^2^ = 2.886, *P* = .410) (Table 4), suggesting the consistent high expression of TAP in different cancers. Our results were in agreement with previous findings that TAP expression occurs in other cancers, including gastric, bladder, and colorectal cancers. Together, it was suggested that TAP expression might serve as a general marker for all kinds of cancers.

**Table 4:**
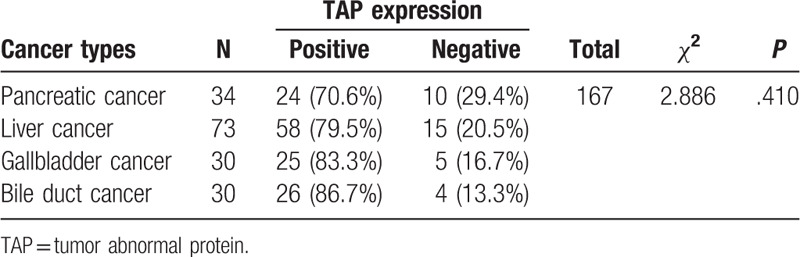
The expression of TAP in subjects with different cancers.

### 3.4 TAP expression was correlated with established cancer marker AFP in liver cancer

Based on the above results, TAP expression was shown to be a general diagnostic indicator for different cancers. To further assess the potential, we compared with the established cancer markers, for example, AFP in liver cancer and CA19-9 in pancreatic cancer. AFP has been used as tumor marker to help detect cancer of the liver and plays important role in the early diagnosis. CA19-9 was found to be a sensitive and specific marker of pancreatic cancer.^[14]^ From our results, it was observed that the expression of TAP was correlated with that of AFP in liver cancer (*P* < .05) (Table 5), suggesting the promise of TAP as an alternative marker for liver cancer. In contrast, no correlation was found between expressions of TAP and CA19-9 in pancreatic cancer (*P* = .241) (Table 6).

**Table 5:**
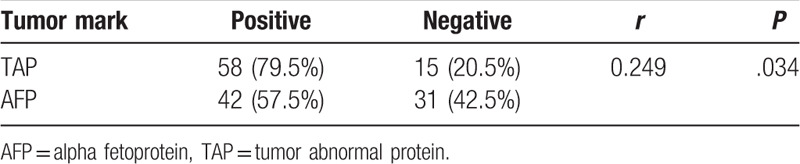
Correlation between TAP and AFP in liver cancer.

**Table 6:**
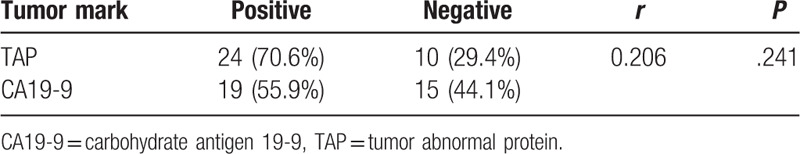
Correlation between TAP and CA19-9 in pancreatic cancer.

**Figure 2:**
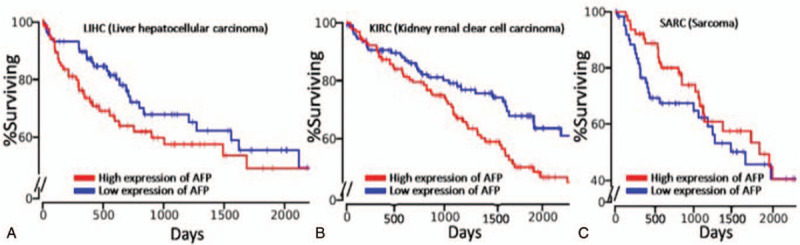
Kaplan–Meier plots for alpha fetoprotein (AFP) expressions in (A) liver hepatocellular carcinoma (LIHC), (B) kidney renal clear cell carcinoma (KIRC), and (C) sarcoma (SARC). The Cancer Genome Atlas (TCGA) database was employed to retrieve the miRNA expression profiles. The upper and lower percentiles were set to 25 for the comparison of Kaplan–Meier plots between different expression levels (high and low) of target genes.

**Figure 3:**
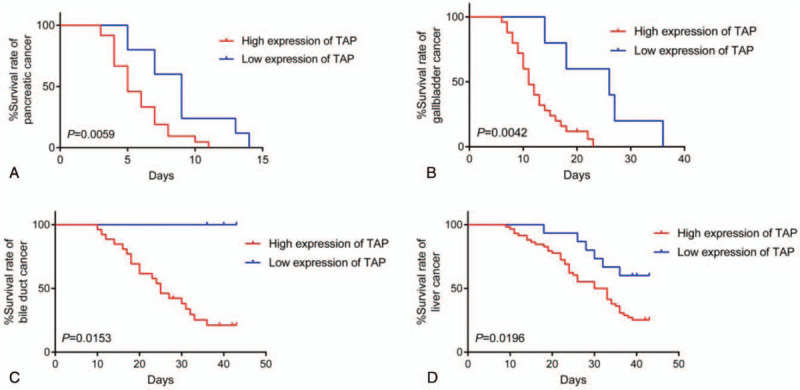
Overall survival of tumor abnormal protein (TAP)-positive and TAP-negative patients with (A) pancreatic cancer, (B) gallbladder cancer, (C) bile duct cancer, and (D) liver cancer.

Moreover, TAP-positive incidence was found to be more frequent than AFP and CA19-9 in liver and pancreatic cancer, respectively (Tables 5 and 6), suggesting the advantage of TAP over these established markers. In addition, most markers were shown to be specific to certain kinds of cancer. For instance, AFP has been proven as a reliable marker of liver cancer, which can be supported by our results (Table 5) as well as the analysis from public database of The Cancer Genome Atlas (TCGA, https://cancergenome.nih.gov/) (Fig. 2). Results showed that high expression of AFP had lower survival rate in patients with liver hepatocellular carcinoma (LIHC) and kidney renal clear cell carcinoma (KIRC). But interestingly, completely opposite result was noticed in sarcoma (SARC). These data clearly indicate the limit of AFP as a potential universal cancer marker. In this study, the overall survival of patients with low expression of TAP in pancreatic, gallbladder, bile duct, and liver cancers was significantly higher than of patients with high expression of TAP (*P* = .0059, *P* = .0042, *P* = .0153, *P* = .0196, respectively) (Fig. 3), suggesting that TAP is a promising indicator in a wide variety of cancers.

### 3.5 TAP expression in preoperative and postoperative patients

We also determined the difference in TAP expression before and after surgery among 51 patients with liver cancer. From our results, it can be seen that the number of patients with TAP-positive expression decreased from 46 to 3 after operation, while TAP-negative expression increased from 5 to 48 (*P* < .001), suggesting the correlation between TAP-positive rate with surgical intervention. Moreover, the TAP value was more closely related to effectiveness of surgery for patients with cancer (Table 7).

**Table 7:**
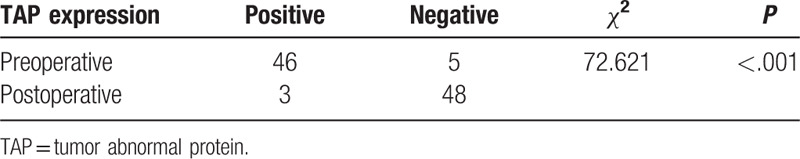
TAP expression in patients with cancer before and after operation.

## 4 Discussion

Early diagnosis and real-time monitoring of cancer recurrence or metastasis are essential for improving the prognosis. Compared with some modern technologies, for example, computed tomography and ultrasonography,^[15,16]^ molecular markers hold advantage in detecting cancer even before the formation of solid tumors.^[17,18]^ However, some established indicators suffer numerous limitations. For instance, AFP has been regarded as a marker for hepatocellular carcinoma but its elevated level was also found in other conditions such as pregnancy, hepatitis, and liver cirrhosis.^[19]^ In addition, as auxiliary diagnosis of pancreatic and gastric cancers, CA19-9 is limited by the relatively low sensitivity and insufficient information.^[20,21]^ Therefore, new indicators with higher sensitivity and specificity for cancer detection are in urgent demand.

In current work, we found consistently high expression of TAP in selected patients with cancer but not healthy ones (*P* < .001). Moreover, there was no significant difference among four different cancers, including liver cancer, pancreatic cancer, gallbladder cancer, and bile duct cancer (*P* > .05). These results were consistent with previous studies that the positive rate of TAP in selected patients with cancer is significantly higher than that of nontumor patients.^[13]^ TAP is reported implicated in the early stage of cancer development,^[22]^ and is able to reflect the quantity and degree of cancerous cells.^[12]^ On these bases, it was suggested that TAP could possibly serve as a universal diagnostic indicator for cancers.

Furthermore, we revealed the positive rate of TAP expression gradually increased as progressing of the disease, which supports the potential role of TAP in the early detection of cancer. Previous reports also suggested TAP might be sensitive to the development of cancer. On this basis, TAP detection holds great promise in identifying the early asymptomatic stage of tumorigenesis for the convenience.^[23]^ Further statistical analysis showed that TAP is characterized by high sensitivity and specificity for the detection of selected cancers, which could be evidenced by a wide variety of indicators, for example, omission diagnostic rate, mistake diagnostic rate, Youden index, positive predictive value, negative predictive value, and overall compliance rate. Notably, we determined the sensitivity of TAP examination for cancer is 78.2% and the specificity is 89.9%, which are in agreement with previous findings that the overall sensitivity and specificity of TAP detection in patients with other different cancers were 85.8% and 80.2%, respectively.^[24]^ Much similar results were also reported by Skowronski et al in digestive tract cancer.^[25]^

Formation of TAP is believed not depend on the tissue origin and histological structure. Therefore, it represents the common material produced in different cancers and has high sensitivity to detect cancer in the early stage. Hence, it can effectively reduce the miss rate by combining with clinical signs and symptoms. In current study, TAP was shown positively correlated with the established marker AFP in liver cancer, confirming its potential role as a promising indicator. Interestingly, the analysis of public TCGA data set showed that high expression of AFP resulted in the significant decrease of cumulative survival among patients with LIHC and KIRC; however, completely opposite change was found in SARC that high expression of AFP caused enhanced cumulative survival, revealing the limitation of AFP that it was only specific to selected (e.g., liver, renal) but not other cancers (e.g., sarcoma). In contrast, consistent performance has been observed for TAP in a variety of different cancers, including gastric, bladder, and colorectal cancers. Besides, this study indicated that high expression of TAP resulted in a significant augment of overall survival among patients with pancreatic, gallbladder, bile duct, and liver cancers. More importantly, TAP was detected more frequently among patients with cancer than other known markers, including AFP and CA19-9, in liver and pancreatic cancer, suggesting the promise of TAP as universal cancer marker. It was also suggested that combination with established markers and comprehensive judgment could improve the accuracy of tumor auxiliary diagnosis.

Surgery is the most effective and common way of treatment of cancer in the clinic, especially in the early stage which was characterized by satisfying therapeutic effect. TAP examination, as simple and nonexpensive means, can be not only used in early diagnosis of cancer but also in postoperative monitoring of the treatment's efficacy. This could be partially evidenced by the observations that the abnormal surface glycosylation is correlated with cancer invasion and metastasis.^[9,26]^ Consistently, our results proved that the expression of TAP in postoperative patients with cancer was significantly lower than that in patients before operation, suggesting the sensitivity of TAP in monitoring the therapeutic effects of surgery. Similarly, Liu and Huang also found TAP is sensitive in monitoring the responsiveness to chemotherapy in patients with advanced gastric or colorectal cancer.^[27]^

In conclusion, for patients with cancer, it is extremely crucial to detect cancer at the very early stage and treat disease as soon as possible. Our findings indicate that TAP detection represents a promising diagnostic tool. However, more extensive studies are in great demand to elucidate the potential role of TAP in cancers.

## Author contributions

**Conceptualization:** Lu-Xi Li, Ruizhi Gong.

**Data curation:** Bin Zhang.

**Formal analysis:** Lu-Xi Li.

**Investigation:** Bin Zhang.

**Methodology:** Lu-Xi Li, Bin Zhang.

**Project administration:** Ruizhi Gong.

**Supervision:** Ruizhi Gong.

**Validation:** Lu-Xi Li, Bin Zhang.

**Writing – original draft:** Lu-Xi Li.
